# Development of a nomogram for predicting positive margins after cold knife conization in patients with high-grade squamous intraepithelial lesions

**DOI:** 10.1097/MD.0000000000042759

**Published:** 2025-06-06

**Authors:** Shuhua Wang, Zhaopeng Ma, Jing Dong, Na Zhang, Xuemei Zhang, Heying Li, Li Chen

**Affiliations:** a Department of Gynecology, Baoding First Central Hospital, Baoding, Hebei, China; b Department of Endocrinology, Baoding First Central Hospital, Baoding, Hebei, China.

**Keywords:** cervical intraepithelial neoplasia, neolpasm residual, nomograms, predictive value of tests, surgical margins, uterine cervical conization

## Abstract

The objective was to develop a nomogram for predicting positive margins after cold knife conization (CKC) in patients with high-grade squamous intraepithelial lesion (HSIL). This retrospective study included patients who underwent CKC at Baoding No. 1 Central Hospital between December 2013 and March 2024. Patients were divided into training (between December 2013 and December 2022) and validation (between January 2023 and March 2024) sets. The least absolute shrinkage and selection operator regression was applied to filter and select relevant variables. Multivariable logistic regression was used for nomogram construction. The model performance was evaluated using various methods, including receiver operating characteristics, decision curve analysis, and calibration analysis. The training and validation sets included 985 and 227 patients, respectively. Age (OR = 1.046, 95% CI: 1.028–1.064, *P* < .001), cervical intraepithelial neoplasia quadrants by punch biopsy (OR = 1.561, 95% CI: 1.348–1.808, *P* < .001), HSIL type (OR = 1.711, 95% CI: 1.102–2.657, *P* = .017), and gland involvement (OR = 1.552, 95% CI: 1.073–2.247, *P* = .020) were associated with positive margins and used for nomogram construction. The predictive model yielded area under the curves of 0.744 and 0.754 in the training and validation sets, respectively. Decision curve analysis indicated a net benefit when using the nomogram, and the calibration curves demonstrated a good fit. This study constructed a nomogram model for predicting positive margins after CKC in patients with HSIL. This nomogram may enable early and accurate patient evaluation, potentially improving clinical outcomes.

## 1. Introduction

Cervical intraepithelial neoplasia (CIN) is a precancerous condition that can lead to cervical cancer and is caused by persistent infection with high-risk human papillomavirus (HPV).^[1]^ The most oncogenic types are HPV-16 and HPV-18.^[2]^ CIN can be categorized into low-grade squamous intraepithelial lesions and high-grade squamous intraepithelial lesions (HSIL).^[3]^ The prevalence of HSIL in Beijing is approximately 70.4 per 100,000 women.^[4]^ Without active treatment, about 30% to 50% of HSIL cases may progress to cervical cancer within 30 years.^[5,6]^ The primary treatment for HSIL is conization, which includes loop electrosurgical excision procedures (LEEP), cold-knife conization (CKC), and laser cone biopsy.^[7,8]^ CKC, serving as both a diagnostic and therapeutic procedure, is widely used due to its simplicity, safety, and effectiveness. However, there is a risk of positive margins after conization, including CKC, indicating the presence of residual precancerous cells. Surgeons typically target macroscopic lesions without removing excessive cervical tissue. A meta-analysis of 44,446 women treated for cervical precancerous lesions revealed an overall positive margin rate of 23.1%, varying by treatment procedure, from 17.8% for laser conization to 25.9% for large loop excision of the transformation zone.^[9]^

The surgical margin status holds significant prognostic value. Indeed, accumulating evidence suggests that women with positive margins after CKC face a higher risk of recurrent CIN and cervical cancer compared to those with negative margins.^[10–13]^ Moreover, managing positive margins after CKC is inconsistent and controversial, especially in patients with fertility considerations.^[14]^ Patients with positive resection margins may require reconization or total hysterectomy, leading to increased economic burden and healthcare resource utilization.^[15]^ In addition, treatments for positive margins, such as adjuvant cervical excision procedures, pose potential issues like the risk of preterm delivery.^[11]^ Hence, there remains a clinical need to identify patients at high risk of positive margins after CKC.

Previous studies have investigated potential risk factors for positive margins after CKC, including age, menopause, histological grade of the cone specimen, depth of conization, and multiple affected quadrants.^[16–19]^ Still, accurately estimating and quantifying the risk of positive margins for individual patients remains challenging and lacks visualization. Therefore, this study aimed to develop a nomogram based on various risk factors influencing the risk of positive margins after CKC.

## 2. Materials and methods

### 2.1. Study design and patients

This retrospective study included patients who underwent conization at the Department of Gynecology, Baoding No. 1 Central Hospital, between December 2013 and March 2024. Patients were divided into a training set (from December 2013 to December 2022) and a validation set (from January 2023 to March 2024). The inclusion criteria were: (i) a preoperative pathological examination of cervical biopsy or cytology indicating HSIL, (ii) postoperative pathology indicating HSIL,^[3]^ (iii) a history of CKC, (iv) clear margin status (positive or negative, without ambiguity), and (v) availability of complete data. The exclusion criteria were: (i) age < 18 years, (ii) diagnosis of invasive cancer at the time of conization (as the focus was on patients with cervical dysplasia), (iii) previous LEEP procedure, (iv) history of HIV or other conditions suppressing the immune system, or (v) immediate hysterectomy. CKC operators: All CKC procedures in this study were performed by 2 senior gynecologists with over 5 years of experience in cervical surgery. To ensure standardization: (i) preoperative colposcopic evaluation was conducted to delineate lesion margins, (ii) the depth and width of the cone were adjusted according to the 2019 ASCCP Consensus Guidelines, (iii) surgical specimens were immediately oriented (at 12, 3, 6, and 9 o’clock) and sent for pathological examination. Pathological examinations: all specimens were independently evaluated by 2 board-certified gynecologic pathologists blinded to clinical outcomes. Diagnostic criteria included: (i) margin status (positive/negative), histological grade (CIN 1/2/3), (ii) presence of microinvasive carcinoma. Discrepancies were resolved by a third senior pathologist. This study was approved by the Ethics Committee of Baoding First Central Hospital (No. [2021]093). As this article is a retrospective study, the Ethics Committee of Baoding First Central Hospital waived the requirement to obtain distinct written informed consent from the patients.

### 2.2. Sample size calculation

We calculated the sample size on the basis of the events per variable metric a widely accepted method instatistical analyses. In our training cohort, the incidence of cognitive impairments 1 month rate of positive margins after CKC was 0.23. Given our intention to include 4 predictor variables and set the events per variable to 10, we calculated the required sample size via the following formula:


$$\begin{array}{l} Sample\;size=\frac{Number\;of\;Variables\;\times \;EPV}{1-Incidence\;\;Rate\;}=\frac{4\;\times \;10}{1-0.23\;}=520 \end{array}$$

### 2.3. Data collection and definitions

The researchers collected information from patient charts on various factors including age, height, weight, body mass index, menopause status, gravidity (≤3 times and ≥4 times), parity (≤1 time and ≥2 times),^[8]^ contact bleeding status, ThinPrep cytology (TCT) data, HPV types before conization, CIN quadrants identified by colposcopy biopsy, HSIL type, gland involvement, and resection scope (depth and width) after cervical conization. These variables were selected based on existing literature^[11,12,20]^ and clinical expertise.

The criteria for positive margins were: (i) pathological findings after cervical conization suggesting the presence of a high-grade cervical intraepithelial lesion at the specimen margin, or (ii) pathological results after cervical conization suggesting that high-grade intraepithelial lesions were very close to the specimen margin (i.e., <1 mm from the specimen margin).^[21]^

### 2.4. Statistical analysis

Statistical analysis was conducted using R 4.3.2 (R Foundation for Statistical Computing, Vienna, Austria). The continuous variables were examined for normal distribution using distribution plots and visual confirmation. The continuous data were expressed as median (Q1 and Q3). The data following a normal distribution were tested using a two-sample *t* test. The data with a skewed distribution were tested using the rank-sum test. The categorical data were expressed as n (%) and tested using Pearson chi-squared test or Fisher exact test, as appropriate.^[22]^ Univariate logistic regression was employed for preliminary variable screening. The least absolute shrinkage and selection operator (LASSO) regression technique was used for data dimensionality reduction and variable selection. Multivariable logistic regression analysis was used to develop a nomogram of positive margins after CKC in patients with HSIL. The discriminatory capacity of the model was determined using receiver operating characteristic curve analysis and the area under the curve (AUC). Internal validation was performed using the bootstrapping method (resampling = 500). The calibration of the model was evaluated using the Hosmer–Lemeshow test, and the clinical utility of the model was assessed by decision curve analysis.^[23]^ External validation was performed using the validation set. Two-sided *P* < .05 was considered statistically significant. This study adhered to the TRIPOD (Transparent Reporting of a multivariable prediction model for Individual prognosis or Diagnosis) statement for reporting.^[24]^

## 3. Results

### 3.1. Basic and clinical characteristics

**Figure 1.**
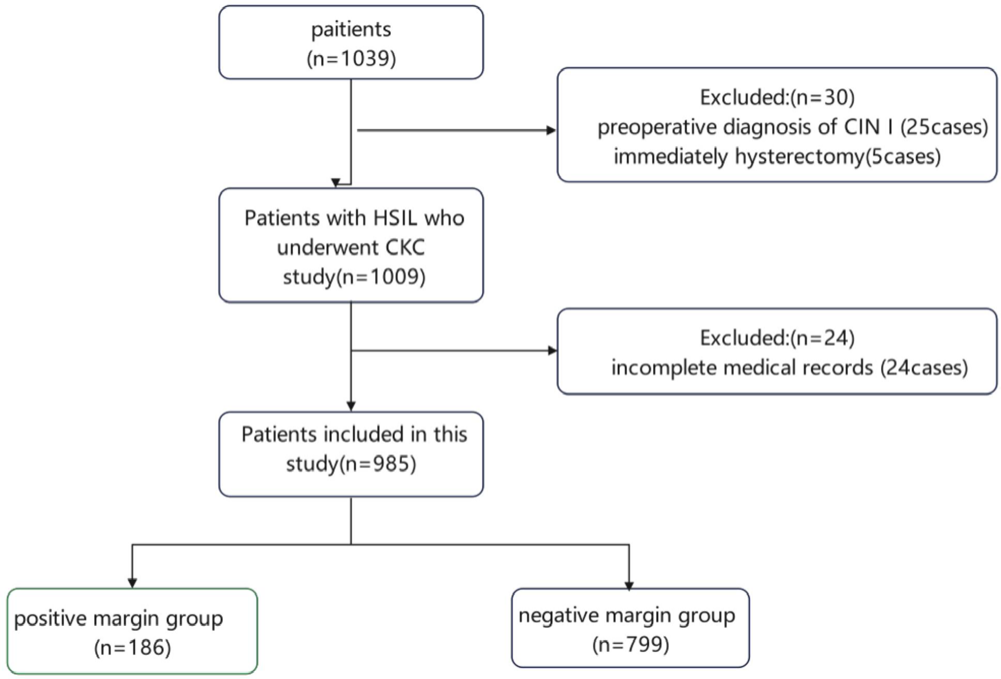
Patient selection flowchart of the training set.

A total of 1039 patients meeting the inclusion criteria were enrolled in the training set. Among them, 54 patients were excluded because of incomplete medical records (n = 24), preoperative diagnosis of CIN 1 (n = 25), or immediate hysterectomy (n = 5) (Fig. 1). Therefore, 985 patients were included for analysis. The median age was 53 years (IQR: 36–51 years; range: 20–73), with 205 (21%) being menopausal. Overall, 367 (37%) and 618 (63%) of patients underwent conization for CIN 2 and CIN 3, respectively (Table 1). In addition, 227 patients were included in the validation cohort.

**Table 1:** Basic and clinical characteristics of the training set.

Variables	Total (n = 985)	Negative (n = 799)	Positive (n = 186)	*P*
Age, median (Q1, Q3)	43 (36, 51)	42 (35, 50)	47 (39, 53)	<.001
BMI, median (Q1, Q3)	23.44 (21.29, 25.78)	23.44 (21.21, 25.71)	23.8 (21.63, 26.12)	.095
Menopause, n (%)	205 (21)	149 (19)	56 (30)	<.001
Gravidity, n (%)				.583
≤3 times	655 (66)	535 (67)	120 (65)	
≥4 times	330 (34)	264 (33)	66 (35)	
Parity, n (%)				.189
≤1 time	437 (44)	363 (45)	74 (40)	
≥2 times	548 (56)	436 (55)	112 (60)	
Contact bleeding, n (%)				.012
No	726 (74)	603 (75)	123 (66)	
Yes	259 (26)	196 (25)	63 (34)	
Cytological grade, n (%)				<.001
No record	35 (4)	26 (3)	9 (5)	
≤LSIL	706 (72)	595 (74)	111 (60)	
≥HSIL	244 (25)	178 (22)	66 (35)	
HPV type, n (%)				.005
Negative	33 (3)	28 (4)	5 (3)	
16/18	628 (64)	489 (61)	139 (75)	
Other high HPV types	264 (27)	232 (29)	32 (17)	
No record	60 (6)	50 (6)	10 (5)	
CIN quadrants by punch biopsy, n (%)				<.001
0	80 (8)	71 (9)	9 (5)	
1	324 (33)	291 (36)	33 (18)	
2	244 (25)	202 (25)	42 (23)	
3	173 (18)	141 (18)	32 (17)	
4	164 (17)	94 (12)	70 (38)	
HSIL type, n (%)				<.001
CIN 2	367 (37)	330 (41)	37 (20)	
CIN 3	618 (63)	469 (59)	149 (80)	
Gland involvement, n (%)	362 (37)	264 (33)	98 (53)	<.001
Cone depth, median (Q1, Q3)	2 (1.5, 2.25)	2 (1.5, 2.28)	1.83 (1.5, 2.18)	.057
Cone width, median (Q1, Q3)	2.5 (2, 3)	2.5 (2, 2.95)	2.5 (2, 3)	.712

BMI = body mass index, LSIL = low-grade squamous intraepithelial lesions.

### 3.2. Risk factors of positive margins in patients with HSIL after CKC

Univariable analysis showed that risk factors for positive margins after cervical conization included age (OR = 2.96, 95% CI: 1.49–5.88, *P* = .002), menopause (OR = 1.88, 95% CI: 1.31–2.69, *P* = .001), contact bleeding (OR = 1.58, 95% CI: 1.12–2.22, *P* = .010), cytological grade (OR = 1.99, 95% CI: 1.40–2.81, *P* < .001), CIN quadrants by punch biopsy (OR = 5.87, 95% CI: 2.75–12.55, *P* < .001), HSIL types (OR = 2.83, 95% CI: 1.93–4.17, *P* < .001), and gland involvement (OR = 2.26, 95% CI: 1.63–3.12, *P* < .001) (Table 2). However, body mass index, gravidity, parity, HPV types, depth of cone, and width of cone did not show significant associations (all *P* > .05).

**Table 2:** Univariable analyses of risk factors associated with positive margins.

Variables	Statistics (n, %)	OR (95% CI)	*P*
Age (years)			
≤29	95 (9.64%)	Reference	/
30–39	305 (30.96%)	1.29 (0.64, 2.61)	.483
40–49	331 (33.60%)	1.69 (0.85, 3.36)	.135
50–75	254 (25.79%)	2.96 (1.49, 5.88)	.002
Menopause			
No	780 (79.19%)	Reference	/
Yes	205 (20.81%)	1.88 (1.31, 2.69)	.001
Gravidity			
≤3 times	655 (66.50%)	Reference	/
≥4 times	330 (33.50%)	1.11 (0.80, 1.56)	.525
BMI	23.76 ± 3.54	1.03 (0.99, 1.08)	.163
Parity			
≤1 time	437 (44.37%)	Reference	/
≥2 times	548 (55.63%)	1.26 (0.91, 1.74)	.163
Contact bleeding			
No	726 (73.71%)	Reference	/
Yes	259 (26.29%)	1.58 (1.12, 2.22)	.010
Cytological grade			
≤LSIL	706 (71.68%)	Reference	/
≥HSIL	244 (24.77%)	1.99 (1.40, 2.81)	<.001
No record	35 (3.55%)	1.86 (0.85, 4.07)	.123
HPV type			
Negative	33 (3.35%)	Reference	/
16/18	628 (63.76%)	1.59 (0.60, 4.20)	.348
Other high HPV types	264 (26.80%)	0.77 (0.28, 2.14)	.620
No record	60 (6.09%)	1.12 (0.35, 3.60)	.849
CIN quadrants by punch biopsy			
0	80 (8.12%)	Reference	/
1	324 (32.89%)	0.89 (0.41, 1.95)	.780
2	244 (24.77%)	1.64 (0.76, 3.54)	.207
3	173 (17.56%)	1.79 (0.81, 3.96)	.150
4	164 (16.65%)	5.87 (2.75, 12.55)	<.001
HSIL type			
CIN 2	367 (37.26%)	Reference	/
CIN 3	618 (62.74%)	2.83 (1.93, 4.17)	<.001
Gland involvement			
No	623 (63.25%)	Reference	/
Yes	362 (36.75%)	2.26 (1.63, 3.12)	<.001
Depth	1.89 ± 0.58	0.78 (0.59, 1.03)	.079
Width	2.46 ± 0.55	1.04 (0.78, 1.39)	.793

BMI = body mass index, CI = confidence interval, LSIL = low-grade squamous intraepithelial lesions, OR = odds ratio.

### 3.3. Nomogram construction and validation

**Figure 2.**
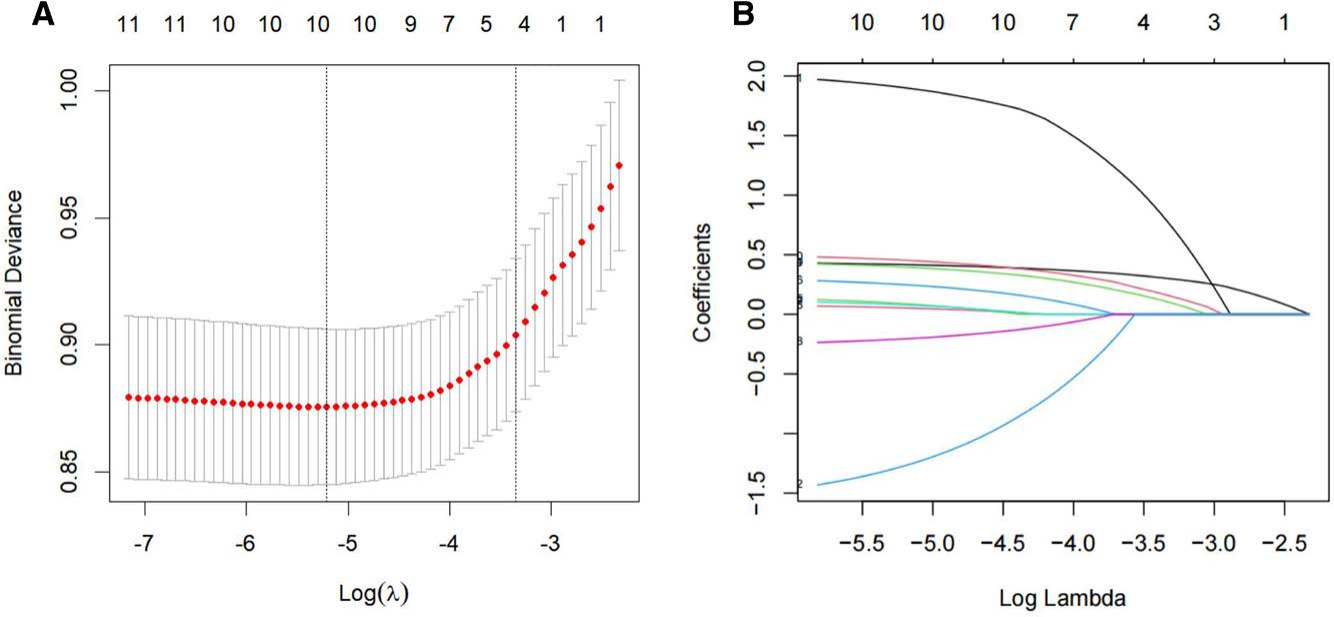
Variable selection using LASSO regression analysis with tenfold cross-validation. (A) Tuning parameter (lambda) selection of deviance in the LASSO regression based on the minimum criteria (left dotted line) and the 1-SE criteria (right dotted line). (B) A coefficient profile plot was created against the log (lambda) sequence. In the present study, the predictor’s selection was according to the 1-SE criteria (right dotted line), where 4 nonzero coefficients were selected. LASSO = least absolute shrinkage and selection operator, SE = standard error.

**Figure 3.**
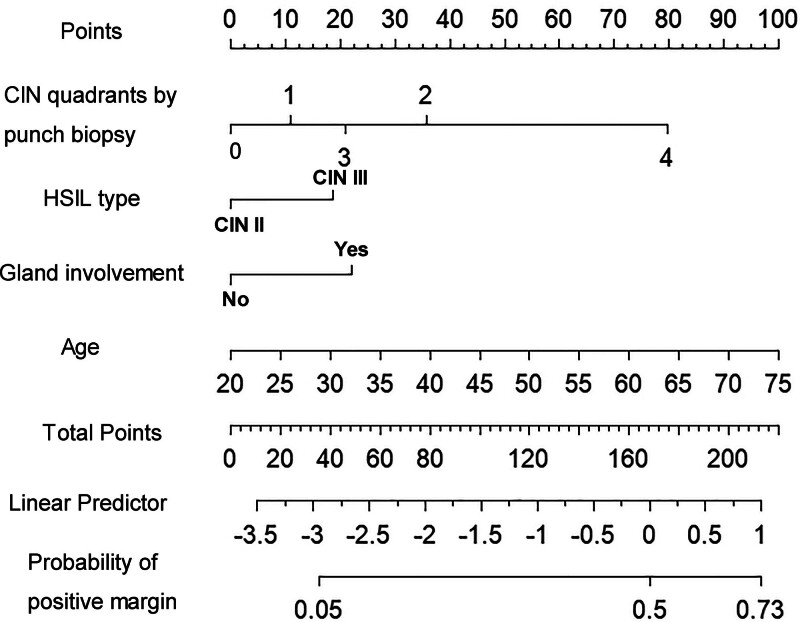
Nomogram for predicting the probability of a positive margin.

Out of the variables collected, 4 were selected based on nonzero coefficients calculated by LASSO regression analysis (Fig. 2). In order to develop a predictive model for positive margins after CKC in patients with HSIL, multivariable logistic regression analysis was performed based on these 4 variables selected by LASSO regression: age (OR = 1.046, 95% CI: 1.028–1.064, *P* < .001), CIN quadrants by punch biopsy (OR = 1.561, 95% CI: 1.348–1.808, *P* < .001), HSIL type (OR = 1.711, 95% CI: 1.102–2.657, *P* = .017), and gland involvement (OR = 1.552, 95% CI: 1.073–2.247, *P* = .020) (Table 3). A nomogram was constructed to present the predictive model (Fig. 3).

**Table 3:** Multivariable analyses of risk factors for patients with positive margins.

Variable	β	OR (95% CI)	*P*
Age	0.045	1.046 (1.028–1.064)	<.001
CIN quadrants by punch biopsy	0.445	1.561 (1.348–1.808)	<.001
HSIL type	0.537	1.711 (1.102–2.657)	.017
Gland involvement	0.440	1.552 (1.073–2.247)	.020

The receiver operating characteristic curves of all variables were presented (Fig. S1, Supplemental Digital Content, https://links.lww.com/MD/P114). The AUC for the predictive model was 0.744, and internal validation using the bootstrap method (resampling = 500) yielded 0.754 (Fig. S2, Supplemental Digital Content, https://links.lww.com/MD/P115). External validation yielded an AUC of 0.954 (Fig. S3, Supplemental Digital Content, https://links.lww.com/MD/P116). Decision curve analysis indicated that the application of this nomogram would provide a net benefit compared to both the treat-all and treat-none strategies (Figs. S4, Supplemental Digital Content, https://links.lww.com/MD/P117 and S5, Supplemental Digital Content, https://links.lww.com/MD/P118). The calibration curve demonstrated a good fit, with the Hosmer-Lemeshow test yielding *P* > .05 (Figs. S6, Supplemental Digital Content, https://links.lww.com/MD/P119 and S7, Supplemental Digital Content, https://links.lww.com/MD/P120).

## 4. Discussion

Cervical conization is an important method for diagnosing and treating cervical lesions. A positive margin after the procedure indicates a higher risk of residual lesions, which may lead to disease recurrence, progression, and affect patient prognosis. Establishing an accurate predictive model for positive margins after cervical conization is crucial for optimizing clinical decision-making and improving patient outcomes.

In this study, a nomogram was developed to predict positive margins after CKC in HSIL patients, using age, CIN quadrants, HSIL type, and gland involvement. The model exhibited good discriminative ability and calibration. Application of this nomogram may aid in clinical decision-making, particularly for identifying individuals at higher risk of positive margins.

Several clinical indicators have been used to predict a positive resection margin in patients with HSIL.^[16–18,20,25]^ In the present study, factors including age, menopause, contact bleeding, TCT results, HPV types, CIN quadrants by punch biopsy, HSIL types before CKC, and gland involvement status were associated with positive margins after CKC. Still, no previous studies have evaluated these variables together to develop a predictive model for positive margins after CKC. This study represents the first attempt to create a visualization of such a predictive model.

Age and menopausal status are significantly associated with an increased risk of predicting a positive cone margin.^[16,17,20]^ Xiang et al^[26]^ evaluated the incidence of positive margins in patients with CIN and microinvasive carcinoma after electrosurgical knife conization; they reported that age 50 years or older (OR = 3.0) and menopausal status (OR = 3.1) were associated with positive margins, supporting the present study. The location of the squamocolumnar junction varies according to age and hormone levels, which could explain why age and menopausal status are associated with margin positivity after cervical conization. The squamocolumnar junction is exocervical in women of childbearing age but often shifts to an endocervical position after menopause,^[16]^ leading to deeper CIN that is not easily exposed, thus increasing the risk of incomplete resection.

The depth of therapeutic conization is an important factor influencing margin involvement. Bae et al^[17]^ reported that patients who underwent conization to a depth > 20 mm were at lower risk for endocervical margin involvement than those who underwent conization to a depth of <20 mm (OR = 0.29). Luca et al^[27]^ suggested that a no <9-mm cone depth provided a fair predictive value in achieving free (endocervical margin) EM. Although depth was associated with positive margins in the univariable analysis, it was not included in the model because it was not identified by the LASSO analysis, which could be related to the sample size and influenced by factors beyond anatomical dimensions, such as tissue friability, surgical technique, or histological grading. Contact bleeding symptoms were also associated with positive margins in the univariable analysis but were not included in the model. Nevertheless, contact bleeding could indicate more advanced lesions even when asymptomatic.

The impact of cytological grade on positive margins after conization is controversial, and previous studies have yielded inconsistent results. Arbin et al^[9]^ showed that an HSIL result on a Papanicolaou smear was advantageous for predicting positive ectocervical margins. In contrast, Ryu et al^[28]^ reported that cytological grade before LEEP was not a significant factor for residual disease. The present study classified TCT results as low-grade squamous intraepithelial lesions and HSIL.^[3]^ The results indicated that HSIL was a risk factor for positive margins. Cytological grade may reflect the severity of cervical lesions. CIN quadrants by punch biopsy, HSIL type, and gland involvement were also significantly associated with positive margins. In the presence of 4 quadrant involvement after cervical biopsy, the likelihood of positive margins significantly increased. The wider the lesion range is, the higher the residual lesion rate is. Tasci et al^[29]^ demonstrated that HSIL involving more than 2 quadrant lesions was a high-risk factor for positive resection margins and residual lesions after conization, supporting the present study. In the meantime, when the cervical biopsy result was CIN 3, the risk of a positive margin after cervical conization was 2.83 times higher than CIN 2. Gland involvement usually suggests deep lesions. Sun et al^[18]^ observed that gland involvement was associated with a positive cone margin in the univariable analysis but not in the multivariable one. In the present study, gland involvement was found to be independently associated with positive resection margins.

Establishing a predictive model allows for the customization of individualized treatment plans: by integrating factors such as patient age, fertility needs, and overall health status, predictive models can assist doctors in formulating treatment plans that are more aligned with the patient’s actual situation. This study focuses on the prediction of positive surgical margins in HSIL patients after CKC, filling a gap in the field left by traditional predictive tools. Although recent AI models (such as Zhang et al^[30]^) have shown excellent performance in complex algorithms, they have mostly focused on disease screening or prognostic stratification (such as HPV typing, recurrence prediction) rather than directly guiding surgical decision-making. The nomogram developed in this study presents in a concise visual form, without the need to rely on complex computing platforms. Clinical doctors can quickly calculate risk scores through patient characteristics (such as age, CIN quadrant), directly guiding preoperative planning (such as adjusting the conization range). Meanwhile, for high-risk populations, postoperative follow-up needs to be strengthened in order to detect cervical cancer as early as possible. However, the key to reducing the incidence of cervical cancer lies in prevention. HPV vaccination has shown significant effects in reducing the incidence of cervical precancerous lesions and cervical cancer. Future cervical cancer management will require more personalized treatment approaches, with therapies increasingly tailored according to the molecular and genetic characteristics of the tumor.^[31,32]^

There are limitations to this study. It included only 985 patients from a single hospital, limiting the generalizability of the results. In addition, the retrospective nature of the study limited the data to those available in the patient charts. This study was cross-sectional and lacked follow-up to analyze HSIL recurrence or cervical cancer development. The nomogram should be validated using a large-scale study.

In conclusion, a nomogram for predicting positive margins after CKC was developed and demonstrated good discrimination and calibration. This nomogram may be useful for counseling women about their risk of positive margins after primary conization. Moreover, it may assist in the clinical management of these patients. Further prospective and external validation of the nomogram is warranted.

## Author contributions

**Conceptualization:** Shuhua Wang, Zhaopeng Ma, Jing Dong, Na Zhang, Xuemei Zhang, Heying Li.

**Data curation:** Shuhua Wang, Zhaopeng Ma, Li Chen.

**Formal analysis:** Shuhua Wang, Jing Dong, Na Zhang, Xuemei Zhang, Heying Li, Li Chen.

**Investigation:** Shuhua Wang, Zhaopeng Ma, Li Chen.

**Methodology:** Shuhua Wang, Zhaopeng Ma, Li Chen.

**Writing – original draft:** Shuhua Wang, Zhaopeng Ma, Jing Dong, Na Zhang, Xuemei Zhang, Heying Li, Li Chen.

**Writing – review & editing:** Shuhua Wang, Zhaopeng Ma, Jing Dong, Na Zhang, Xuemei Zhang, Heying Li, Li Chen.

## Supplementary Material


