# Supplementary figures and images for: Development of a nomogram for predicting positive margins after cold knife conization in patients with high-grade squamous intraepithelial lesions
Source: Medicine (Baltimore). 2025 Jun 6;104(23):e42759. doi: 10.1097/MD.0000000000042759 (PMC12150994; doi:10.1097/MD.0000000000042759)

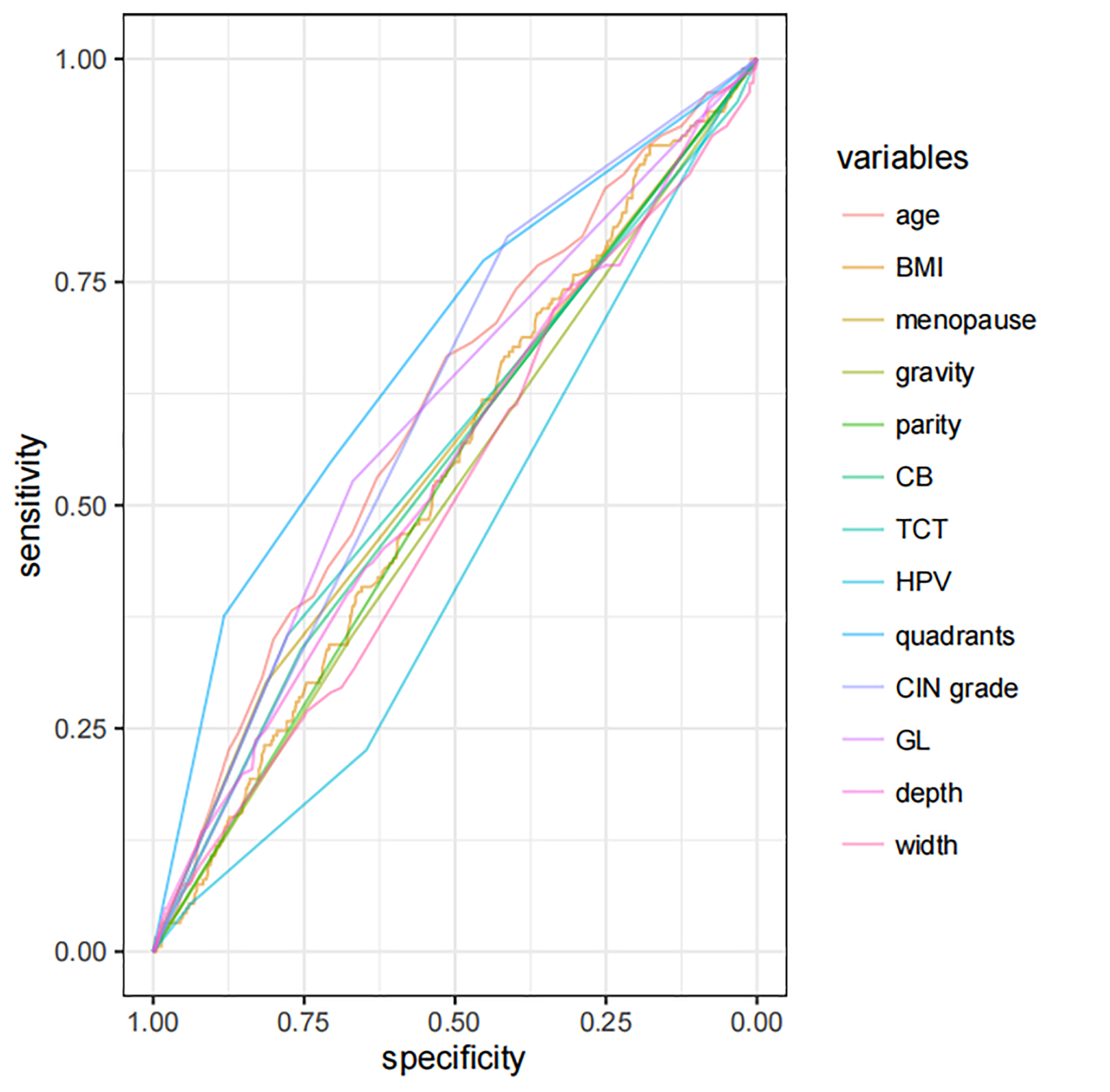

Supplement: Supplementary file 1 [file medi-104-e42759-s001.tif]

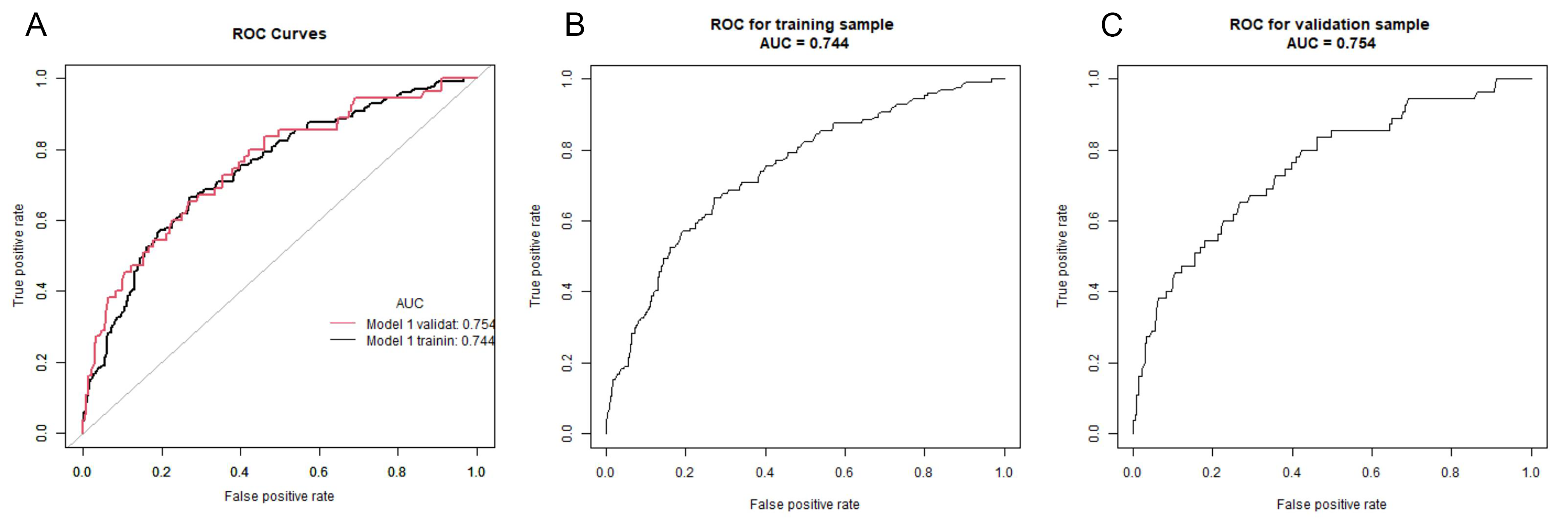

Supplement: Supplementary file 2 [file medi-104-e42759-s002.tif]

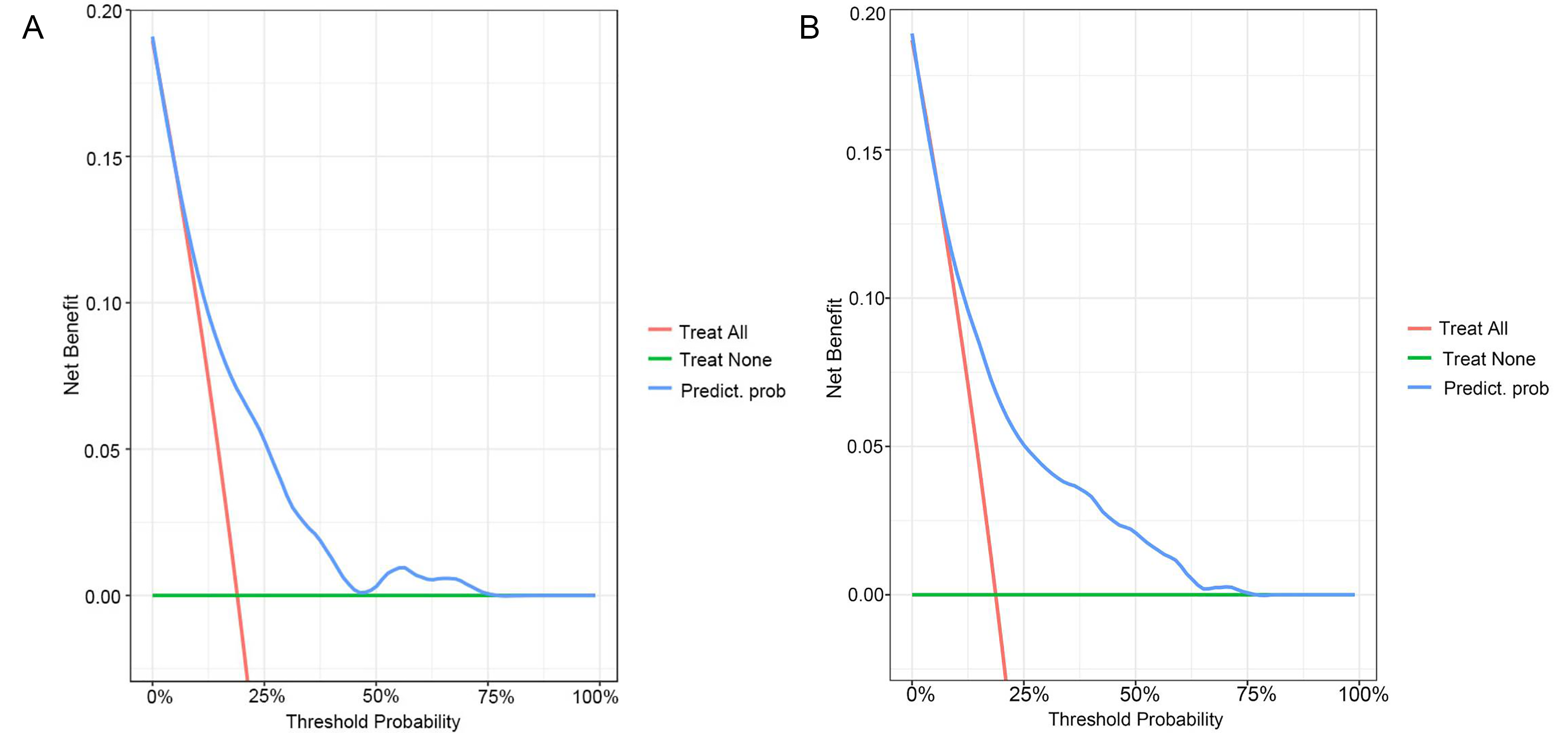

Supplement: Supplementary file 4 [file medi-104-e42759-s004.tif]

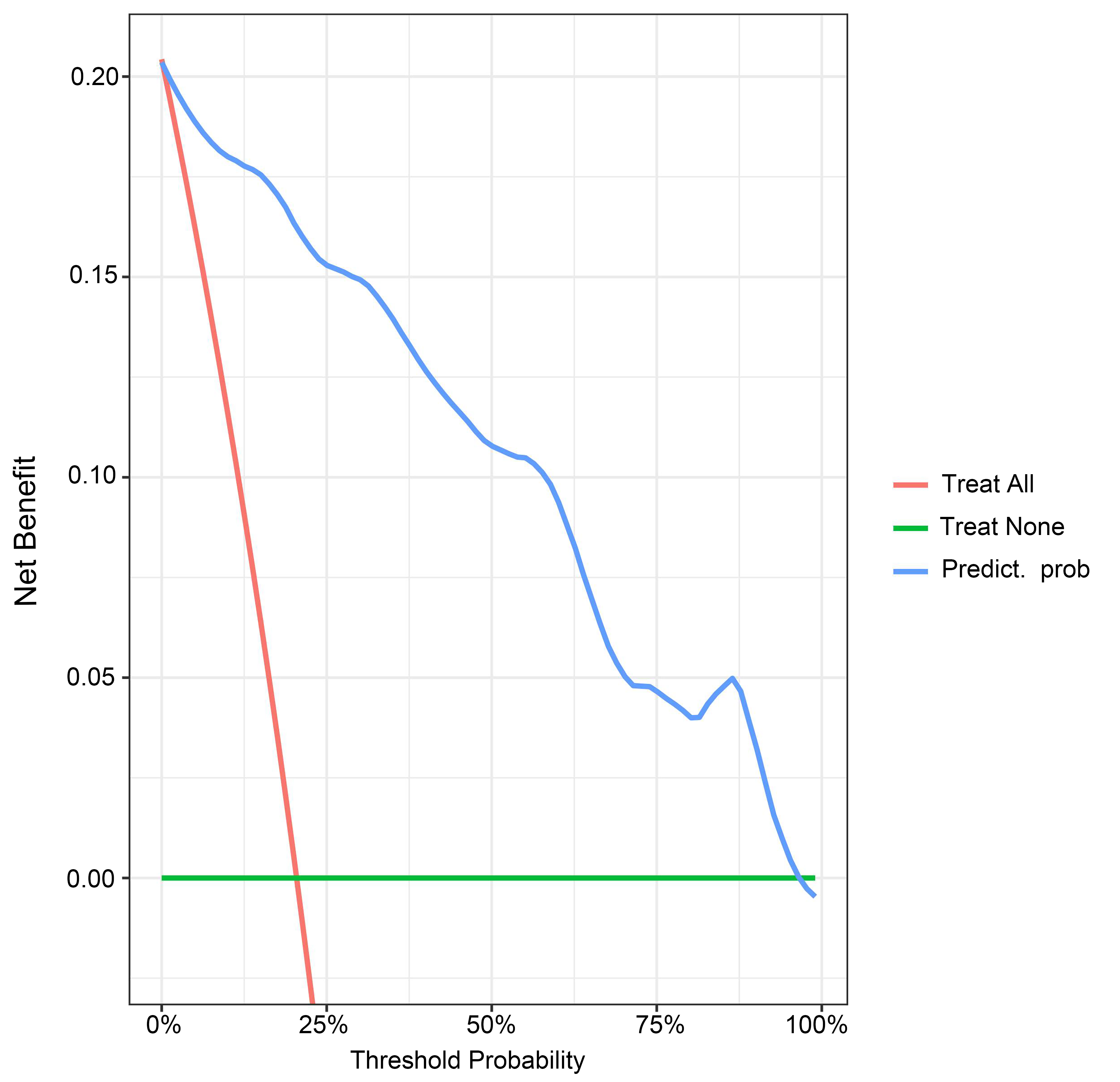

Supplement: Supplementary file 5 [file medi-104-e42759-s005.tif]

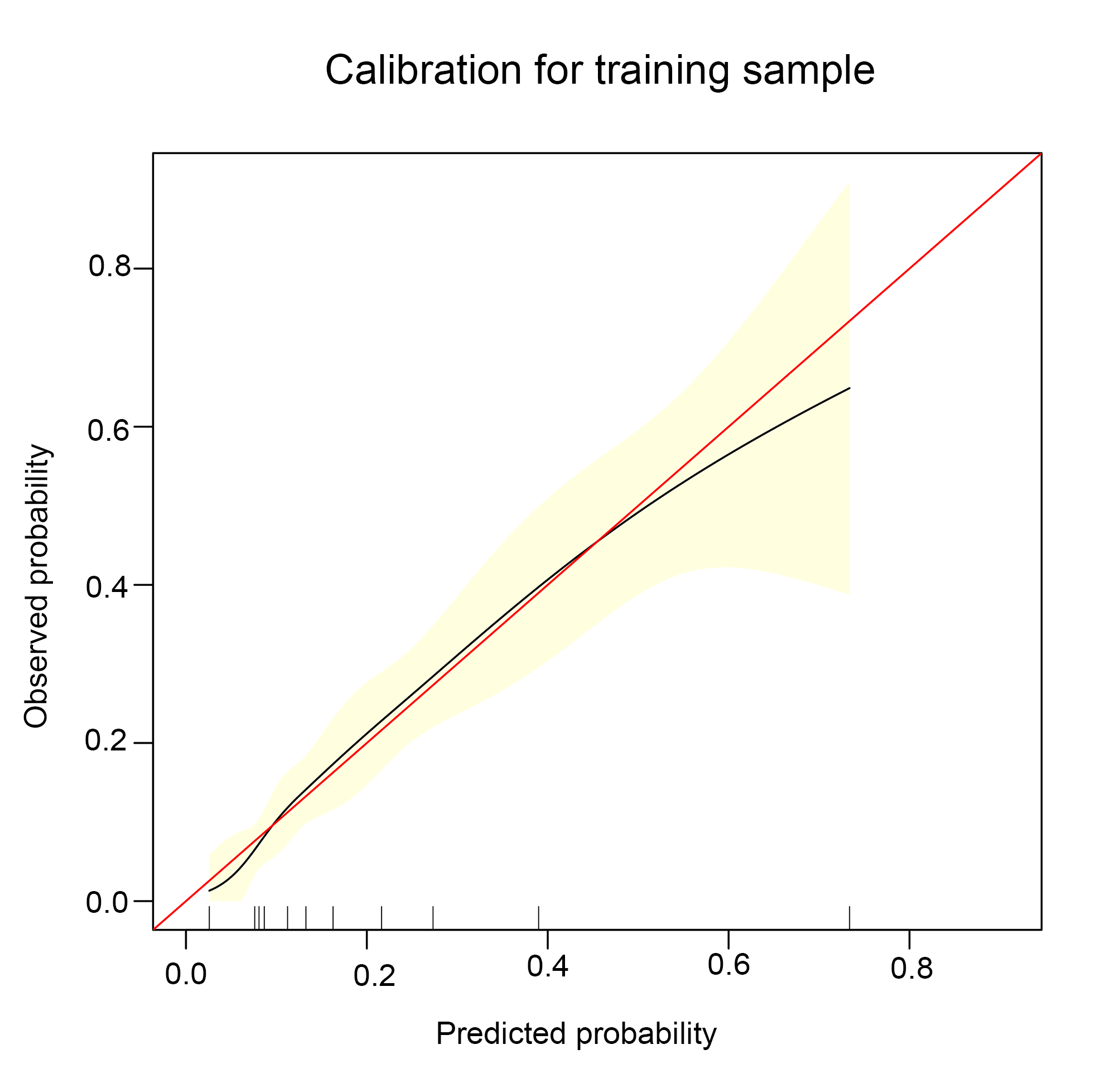

Supplement: Supplementary file 6 [file medi-104-e42759-s006.tif]

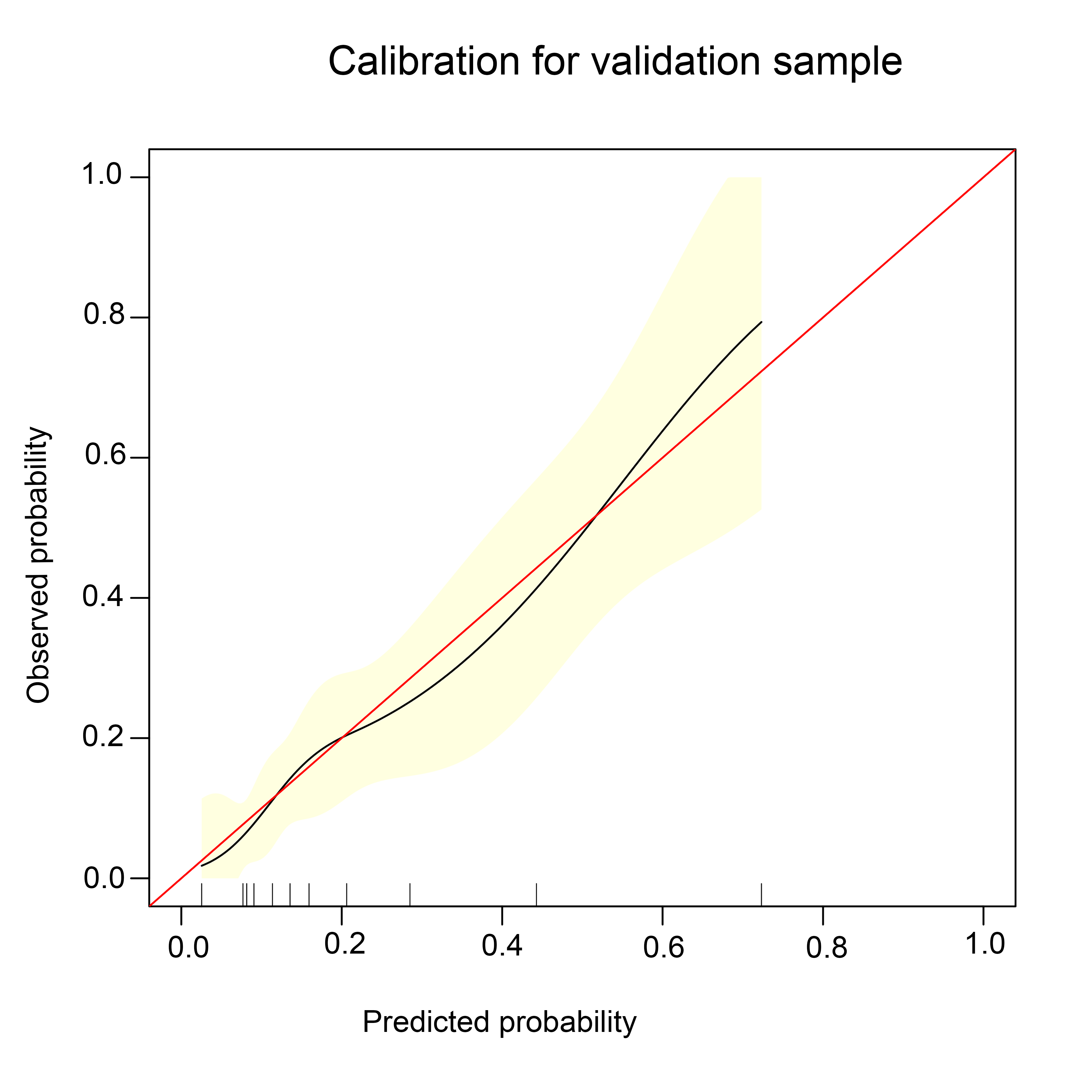

Supplement: Supplementary file 7 [file medi-104-e42759-s007.tif]
